# IgG4-associated orbital and ocular inflammation

**DOI:** 10.1186/s12348-015-0047-y

**Published:** 2015-05-29

**Authors:** Cecilia S Lee, George J Harocopos, Courtney L Kraus, Aaron Y Lee, Gregory P Van Stavern, Steven M Couch, P Kumar Rao

**Affiliations:** Department of Ophthalmology and Visual Sciences, Washington University in St. Louis, CB 8096, 660 S. Euclid, St. Louis, MO 63110 USA; Department of Ophthalmology, University of Washington, Seattle, WA USA; Department of Pathology and Immunology, Washington University in St. Louis, St. Louis, MO USA; Department of Ophthalmology, Wilmer Eye Institute, Johns Hopkins University, Baltimore, MD USA; Department of Ophthalmology, University of British Columbia, Vancouver, BC Canada

**Keywords:** IgG4-associated ocular inflammation, IgG4-associated orbital inflammation, Histopathology

## Abstract

**Background:**

IgG4-associated orbital and ocular inflammation is a relatively unknown entity characterized by sclerosing inflammation with infiltration of IgG4-positive plasma cells. Some so-called idiopathic inflammation syndromes are being re-classified as IgG4-associated inflammation with histopathologic evaluation.

**Findings:**

We report three cases with differing manifestations of IgG4-associated ocular and orbital inflammation: a case of recurrent, treatment-refractory sclero-uveitis that was diagnosed as granulomatosis with polyangiitis with an IgG4-related component, a case of pachymeningitis with optic neuritis that resulted in permanent visual loss, and a case of orbital inflammatory pseudotumor. All three would have been incompletely diagnosed without thorough histopathologic evaluation (including immunohistochemistry).

**Conclusions:**

IgG4-associated disease is an idiopathic, multi-organ inflammatory state that can manifest as chronic, relapsing, sclerosing inflammation in virtually any organ system. There is a wide range of presentations in ocular and orbital inflammation. Ophthalmologists should keep IgG4-associated inflammation in mind when examining chronic, sclerofibrosing inflammation with multi-system involvement. The histology of biopsy specimens is crucial in making the correct diagnosis. Timely assessment may lead to fewer diagnostic tests and more targeted therapy.

## Findings

IgG4-associated disease is a relatively newly discovered entity characterized by sclerosing inflammation, a lymphoplasmacytic infiltration full of IgG4-positive plasma cells and frequently associated with elevated serum IgG4 concentrations [1]. It was first described as a systemic condition in 2003 when extra-pancreatic manifestations were identified in patients with autoimmune pancreatitis, a condition known to be associated with elevated levels of IgG4 [2]. Since that discovery, the presence of IgG4-related inflammation has been detected in virtually all organs, particularly the pancreas, liver, kidney, lung, and thyroid [1, 3, 4, 5]. The histopathological features exhibit remarkable consistency across organ systems. In ophthalmology, IgG4-positive sclerosing inflammation most frequently presents with involvement of the orbit/lacrimal gland [1, 5, 6], but recent case reports have described conjunctival and scleral involvement [3, 7]. We report a series of three heterogeneous patients including some unusual presentations of IgG4-associated ocular/orbital inflammatory disease: sclero-uveitis, pachymeningitis with associated bilateral optic neuropathy/perineuritis, and inflammatory pseudotumor of the orbit.

### Case summaries

#### Case 1

**Fig. 1:**
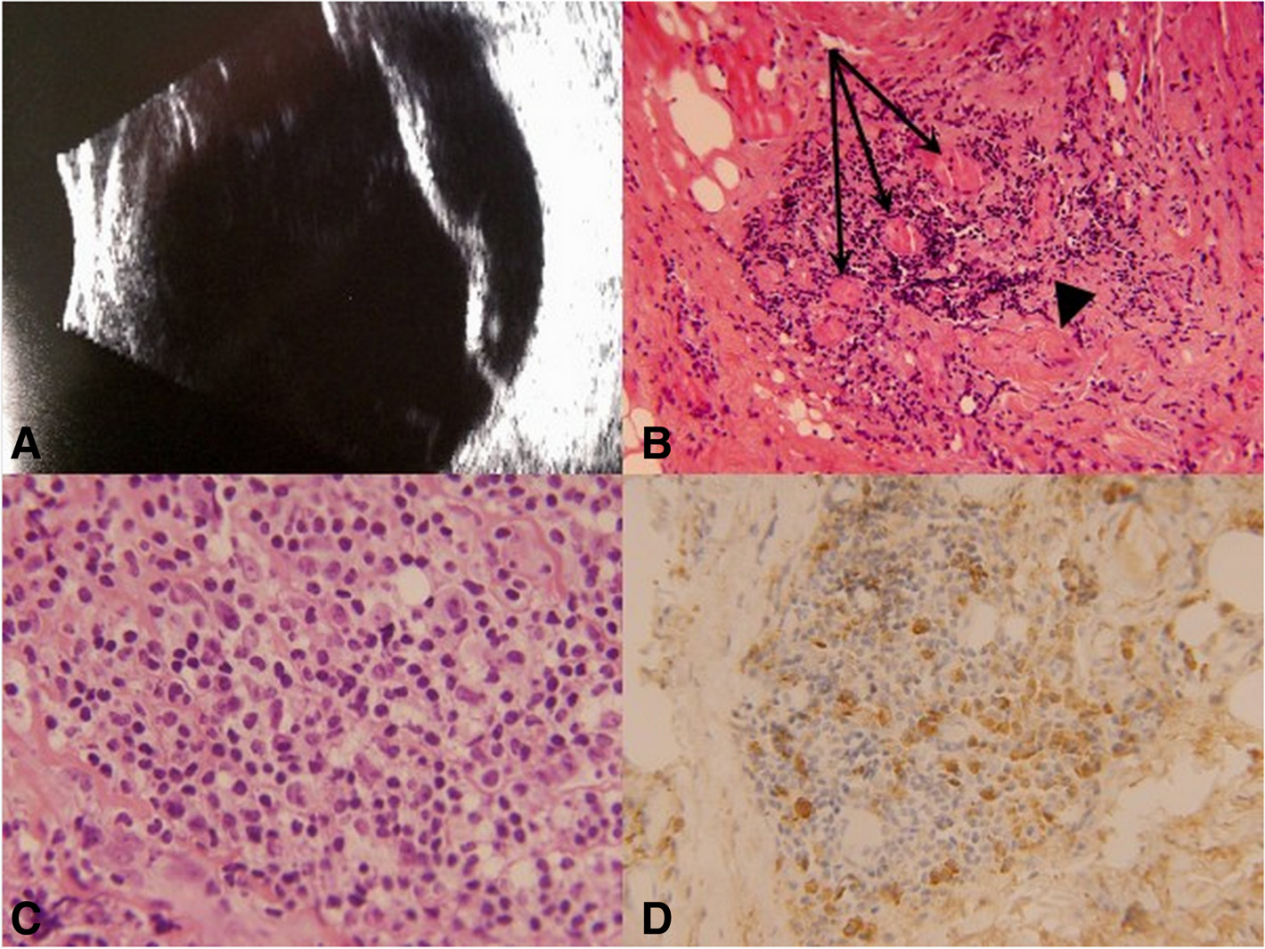
**a** B-scan ultrasonography shows serous elevation of the retina in the right eye of case 1. **b–d** Histology of episcleral/orbital biopsy from case 1. **b** Chronic inflammation in a background of fibrosis is seen. In this field, there is a vasculo-centric component to the inflammation (*arrows*), along with a hyalinized vessel (*arrowhead*) (H&E stain, original magnification, ×200). **c** Higher magnification of another region exhibits mixed chronic inflammatory cells, including lymphocytes, plasma cells, and macrophages (H&E stain, original magnification, ×600). **d** IgG4 immunostain was positive in up to 50 plasma cells per high-power field, comprising over 50 % of the total plasma cell population (original magnification, ×400)

A 79-year-old woman was referred to our institution with a 1-year history of tearing, blurry vision, pain, photophobia, and redness in her right eye. Past medical history was significant for breast cancer, which required right lumpectomy 14 years prior and left lumpectomy plus radiation that was completed about 1 year prior to referral. The patient’s symptoms in the right eye had begun about 1 month following the completion of the radiation treatment for breast cancer, at which point she was diagnosed with nodular scleritis inferotemporally in the right eye, and she was found to have elevated perinuclear accentuated anti-neutrophil cytoplasmic antibodies (p-ANCA), leading to suspicion for granulomatosis with polyangiitis. She was managed by multiple ophthalmologists and rheumatologists. Medical history also included insulin-dependent diabetes, hypertension, hyperlipidemia, anemia, and colon cancer about 16 years prior. There was a history of maxillary and sphenoid sinusitis, for which the patient had right mastoid surgery, as well as a history of bilateral ear tubes. The patient also had, at the time of presentation, bilateral renal oncocytomas treated with cryoablation, but renal function was normal. Past ocular history included open-angle glaucoma initially well-controlled with topical therapy alone but later exacerbated by an inflammatory/corticosteroid-induced component in the right eye. Prior to referral, the patient had been treated with oral prednisone, with good response, but experienced recurrences whenever the dose was tapered. She had also been treated with cyclophosphamide and indomethacin but had a history of poor response to azathioprine. Upon referral to our institution, best corrected visual acuity (BCVA) was 20/80 in the right eye. Slit-lamp examination demonstrated 360° of conjunctival and scleral injection, nodules in the temporal sclera, and 1+ nuclear sclerotic cataract. Dilated fundus examination and B-scan ultrasound showed panuveitis and serous retinal detachment associated with subretinal fluid (Fig. 1). A “T” sign was also noted on ultrasound, consistent with posterior scleritis. Left eye exam was unremarkable. The differential diagnosis included inflammatory sclero-uveitis and intraocular lymphoma but favoring a diagnosis of inflammatory anterior/posterior scleritis and uveitis. An extensive work-up for sclero-uveitis was only positive for p-ANCA with a titer of >1:640 (normal <1:10). Pertinent negatives included rheumatoid factor, rapid plasma reagin, fluorescent treponemal antibody, and angiotensin converting enzyme. The leading diagnosis was granulomatosis with polyangiitis, and the patient was treated with oral prednisone, cyclophosphamide, and indomethacin and showed clinical improvement, with vision improving to 20/40.

Nine months later, our patient was referred again with an exacerbation of her inflammation after cataract surgery in the right eye (performed at an outside institution). Her BCVA was 20/200. Slit-lamp examination showed mild scleral injection with a fibrotic-appearing lesion adherent to the medial sclera. Dilated fundus examination demonstrated chorioretinal scars with subretinal fibrosis and pale optic nerve. The infiltrative fibrotic lesion on the sclera was biopsied, and histologic examination revealed mixed chronic inflammatory infiltrate with fibrosis consistent with orbital inflammatory pseudotumor (Fig. 1b–d). Although no actively necrotizing vasculitis was seen, a portion of the inflammation exhibited a vasculo-centric pattern, with hyalinization of vessels, raising the possibility that these findings represented the late stage of a vasculitic process, and accordingly, granulomatosis with polyangiitis remained in the differential diagnosis (Fig. 1b, c).

**Fig. 2:**
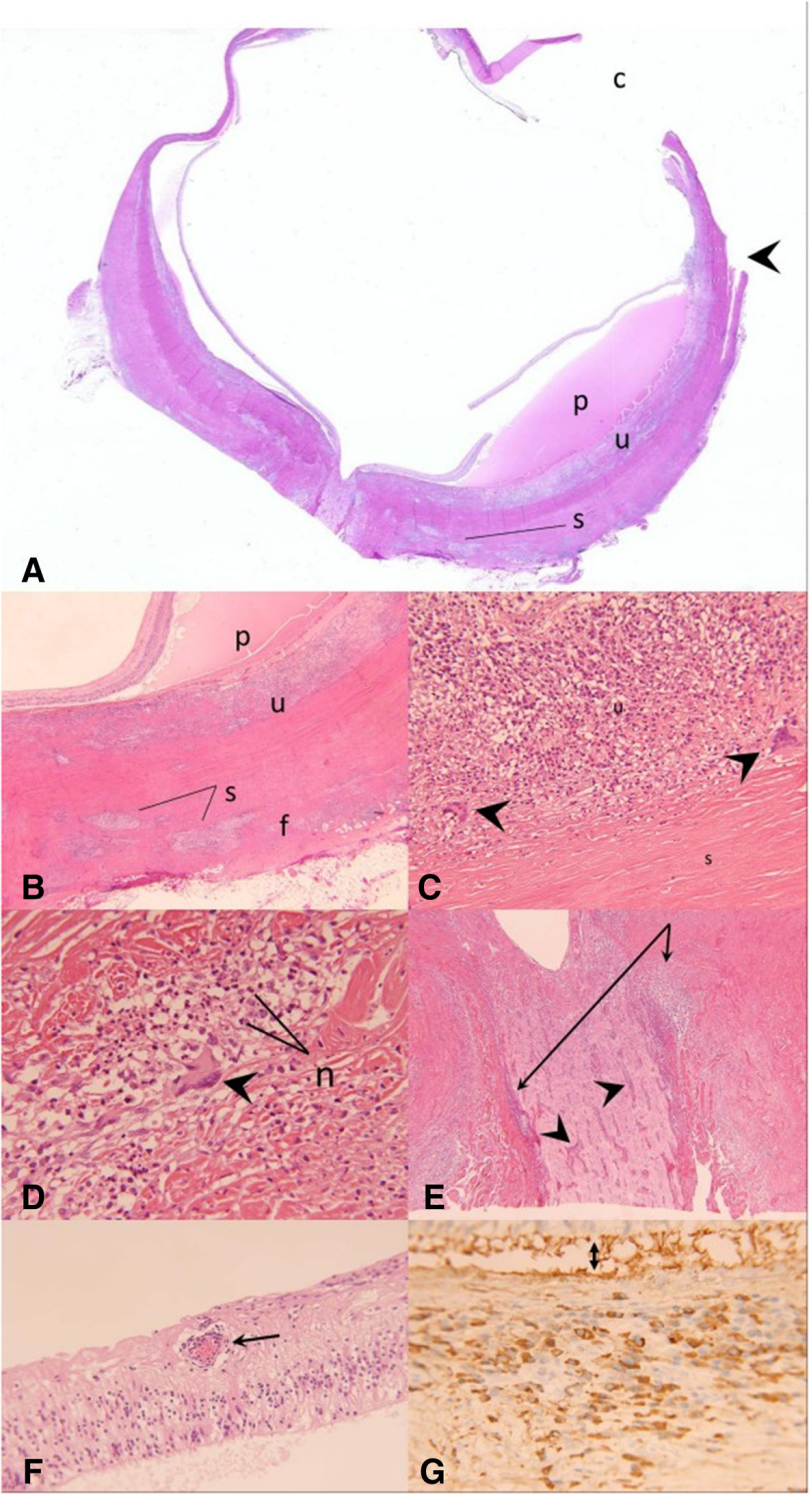
Histology of enucleated globe from case 1. **a** At low power, serous detachment with subretinal protein (*p*), uveitis (*u*) with choroidal thickening, and scleritis (*s*) with scleral/episcleral thickening are appreciated. The abrupt defect in the episcleral fibrotic plaque medially corresponds to the prior episcleral/orbital biopsy site (*arrowhead*). Optic nerve cupping is also evident. The gap in the eye wall where the cornea would ordinarily be (*c*) is artifactual (H&E stain, original magnification, ×1). **b** Higher magnification of same region of globe medially that was labeled in **a**. Besides the previously mentioned findings, episcleral/scleral fibrosis (*f*) is appreciated surrounding the pockets of inflammation, corresponding to the fibrotic-appearing epsicleral/scleral plaque that was noted clinically (H&E stain, original magnification, ×20). **c** Higher magnification reveals granulomatous uveitis (*u*), with several giant cells visible in the field (*arrowheads*). Sclera (*s*) is visible at the bottom of the field (H&E stain, original magnification, ×200). **d** Higher magnification of sclera reveals necrotizing and granulomatous scleritis, with both neutrophils (*n*) and macrophages including a giant cell (*arrowhead*) visible in the field (H&E stain, original magnification, ×400). **e** Higher magnification of optic nerve, revealing peri-neuritis/neuritis, with chronic inflammatory cells infiltrating both the optic nerve sheath (*arrows*) and pial septa within the optic nerve (*arrowheads*). Optic nerve cupping/atrophy is also seen (H&E stain, original magnification, ×40). **f** Higher magnification of the retina reveals severe atrophy and exhibits the chronic vasculitis that was noted in some of the retinal vessels (*arrow*) (H&E stain, original magnification, ×200). **g** IgG4 immunostain highlights >70 plasma cells per high-power field, corresponding to >50 % of the plasma cell population, in the uvea and sclera/episclera. The uvea is captured in this field. The *double-headed arrow* delineates the subretinal space (original magnification, ×400)

Treatment subsequently included methotrexate and infliximab infusions, but the patient continued to have recurrent eye pain and eventually developed a blind, painful right eye with end-stage glaucoma and persistent vitritis, besides evidence of optic nerve sheath inflammation on imaging, leading to an enucleation. The histopathology of the globe showed necrotizing and fibrotic granulomatous scleritis and granulomatous panuveitis (Fig. 2). Of note, microbial stains were negative. Chronic optic neuritis was also noted, with optic cupping and atrophy (Fig. 2a, e). There was serous detachment of the retina with subretinal protein, correlating with the clinical appearance (Fig. 2a). The retina exhibited severe atrophy, with chronic vasculitis seen in some of the retinal vessels (Fig. 2f), and a neovascular epiretinal membrane was appreciated overlying the pars plana. There were no atypical lymphocytes or any evidence of lymphoma on immunohistochemistry, with T cells (CD3-positive) far outnumbering the B cells (CD20-positive), and with *in situ* hybridization demonstrating a relatively even mixture of kappa- and lambda-positive plasma cells (not shown). Many of the plasma cells stained positive with IgG4 (Fig. 2g), i.e., about 70 per high-power field, comprising over 50 % of the total plasma cell population. (The total plasma cells were assessed with CD138 immunostain, not shown.) The previous episcleral/orbital biopsy was then retrospectively also stained with IgG4 immunostain, which likewise revealed positivity in >50 % of the plasma cell population (Fig. 1d). (Serum IgG4 was not obtained.)

#### Case 2

**Fig. 3:**
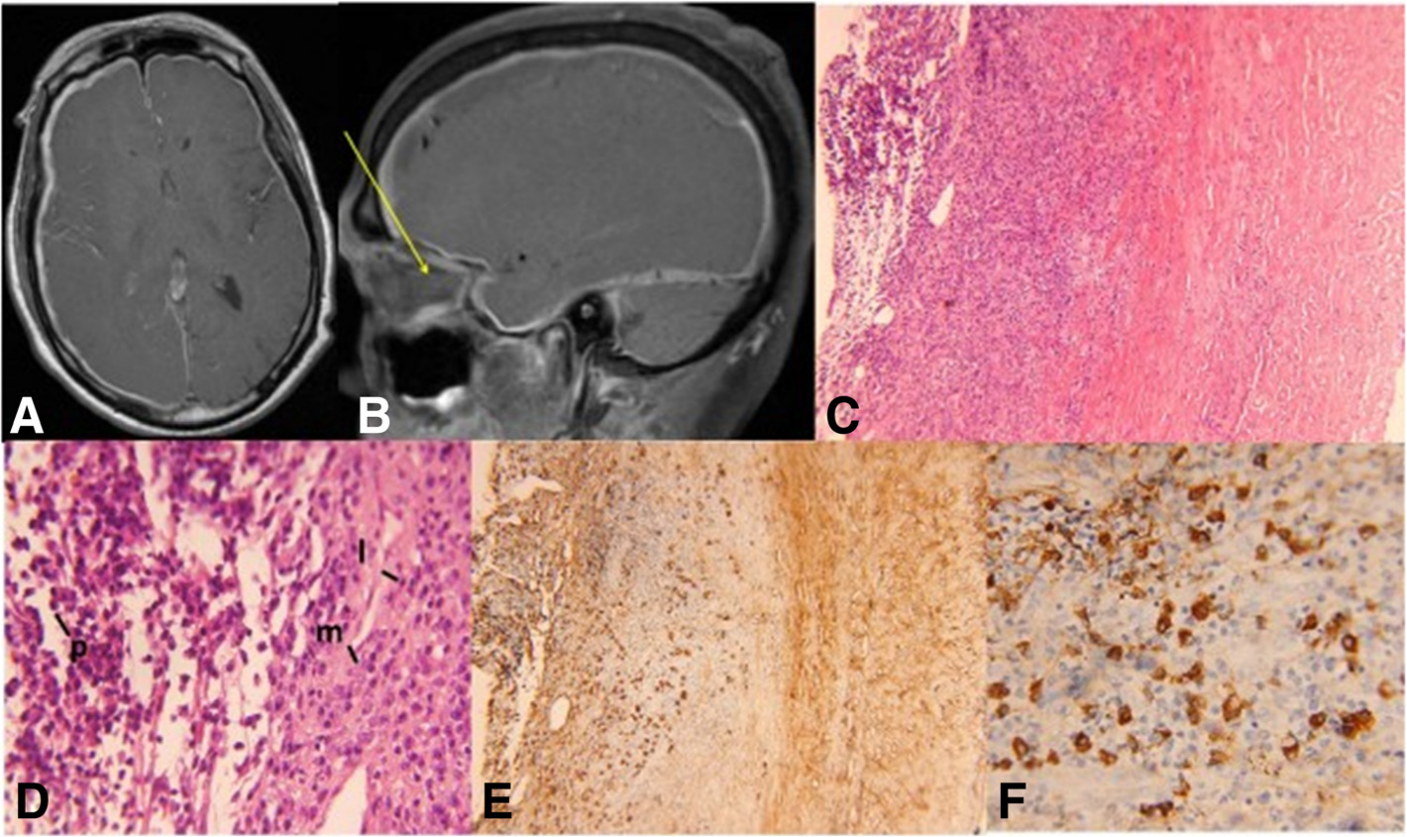
Neuroimaging and histopathology of Case 2. **a** T1 axial post-contrast image shows diffuse meningeal enhancement. **b** T1 post-contrast sagittal image shows possible enhancement along right optic nerve sheath (*arrow*). **c** Histologic section of meninges, with arachnoid portion on the left and dura on the right. A chronic inflammatory infiltrate is concentrated in the leptomeninges (H&E stain, original magnification, ×100). **d** Higher magnification exhibits a mixed chronic inflammatory infiltrate, including abundant plasma cells (*p*), as well as lymphocytes (*l*) and macrophages (*m*) (H&E stain, original magnification, ×400). **e** IgG4 immunostain highlights numerous plasma cells (original magnification, ×100). **f** Higher magnification reveals >40 IgG4-positive plasma cells per high-power field, comprising about 40 % of the total plasma cell population (original magnification, ×400)

A 54-year-old man with a 4-month history of recurrent sinusitis, 100-lb unintentional weight loss, gait instability, and generalized weakness presented with headache and painless, bilateral visual loss. Bilateral temporal artery biopsies were performed and showed no arteritis. Magnetic resonance imaging (MRI) of the brain revealed mild meningeal enhancement and a chronic subdural hematoma causing mild midline shift. Following surgical drainage, visual acuity remained unchanged, and his headaches worsened. Pertinent clinical findings were no light perception in both eyes, amaurotic pupils, and bilateral mild, temporal optic disc pallor. Ocular coherence tomography (OCT) of both optic nerves demonstrated normal nerve fiber layer thickness. Laboratory tests failed to suggest an etiology of his vision loss. Lumbar puncture showed lymphocytic pleocytosis but cytology and flow cytometry were negative for lymphoma. Repeat brain MRI was performed and demonstrated diffuse pachymeningeal enhancement (Fig. 3a) with possible involvement of the optic nerve sheath (Fig. 3b); a dural biopsy was performed.

Histopathology demonstrated a dense infiltrate of lymphocytes and plasma cells, with no definite vasculitis or granulomatous inflammation (Fig. 3c, d). The possibility of marginal zone lymphoma was considered but was ruled out by immunohistochemistry, with T cells (CD3-positive) outnumbering B cells (CD20-positive), and with *in situ* hybridization revealing a kappa:lambda ratio of <4:1 among the plasma cells (not shown). *In situ* hybridization for Epstein-Barr virus was negative (not shown). Additionally, molecular genetic studies did not show any evidence of a clonal IgH gene rearrangement by polymerase chain reaction (PCR). However, IgG4 immunostain was markedly positive, with 40 IgG4-positive cells per high-power field (HPF), comprising over 40 % of the total plasma cell population (Fig. 3e, f). (The total plasma cell population was assessed with CD138 immunostain, and IgG immunostain was also obtained, showing that all IgG-positive cells were also IgG4-positive, i.e., IgG4:IgG ratio of 1, not shown). Subsequent bone marrow biopsy also showed no evidence of lymphoma (small CD19-positive B-cell population with normal kappa:lambda light chain ratio), and fine-needle aspiration biopsy of a left supraclavicular lymph node showed similar findings negative for lymphoma.

On his systemic work-up, p-ANCA was borderline positive, and serum IgG showed elevated IgG4 subclasses (148.0; normal range 2.4–121.0). Accordingly, the diagnosis of IgG4-related meningeal disease was made. The patient was started on aggressive immunosuppression with high-dose corticosteroids to be followed by either cyclophosphamide or rituximab. However, due to insurance issues, he was able to receive only methotrexate. There was near-total resolution of meningeal enhancement on repeat MRI, but his visual acuity improved to only bare light perception in both eyes. He remains stable on methotrexate almost 1 year from his diagnosis.

#### Case 3

A 76-year old man with a history of bilateral lacrimal gland masses and an otherwise unremarkable ocular examination underwent left orbital biopsy at another institution. Clinical suspicion was high for possible lymphoma due to the additional presence of cervical and axillary lymphadenopathy. The histologic diagnosis at that time was reactive lymphoid hyperplasia with fibrosis. Immunohistochemistry, kappa/lambda *in situ* hybridization, and flow cytometry were negative for lymphoma. The patient was lost to follow-up without any further work-up. He presented a year later due to recurrence of orbital inflammation, at which point the surgeon requested histologic work-up for IgG4-related disease. The slides from the prior orbital biopsy were requested and reviewed, showing lymphoid follicles with intervening bands of fibrosis and mixed chronic inflammatory cells. The tissue block was also received, and additional sections were cut for CD138 and IgG4 immunostaining. There were at least 150–180 IgG4-positive plasma cells per high-power field, comprising over 40 % of the total plasma cell population (as assessed by CD138). (Serum IgG4 was not obtained.) This was considered consistent with IgG4-associated orbital inflammatory pseudotumor with sclerosing features. The patient was initially treated with prednisone and then transitioned to intravenous rituximab as first-line therapy and had an excellent response with no recurrence at 2 years following therapy.

### Discussion

IgG4-associated disease is a relatively newly described, heterogeneous entity, initially described in autoimmune pancreatitis. Hamano et al. found elevated serum IgG4 level in patients with autoimmune pancreatitis, and since then, IgG4 has been used as an important marker to diagnose and re-classify select disease states previously regarded as idiopathic [8]. The disease is characterized by IgG4-positive plasmacytic infiltrate, fibrosis of the involved organs, and often elevated serum IgG4 level. Histologic analysis of biopsy specimens is critical in diagnosing this condition [1]. IgG4-related disease tends to affect people in the middle-aged to elderly years. The most commonly involved sites are the pancreas, the salivary and lacrimal glands, and the orbit [9]. However, the list of involved organs continues to grow with increasing awareness.

Immune disregulation has been hypothesized as the most probable mechanism of IgG4-associated disease [9]. IgG4 is only a minor component of four subsets of IgG. The hypothesized pathogenesis involves upregulated expression of T-helper 2 cells; cytokines such as interleukin-4,5, and 13; and regulatory cytokines such as interleukin-10 and transforming growth factor-beta [8, 10]. It has been suggested that this upregulation could be due to repeat, prolonged exposure to specific antigens. It is interesting to note that up to 40 % of patients with IgG4-related disease suffer from allergic diseases such as asthma or chronic sinusitis [11].

The treatment is mainly with oral corticosteroids, to which the inflammation is generally exquisitely responsive; nevertheless, patients remain on long-term steroid therapy, and relapse is common after cessation of corticosteroids [12]. Other therapies such as radiation or immunomodulating agents are not well-described in the literature [6]. A recent prospective, open-label trial of 30 patients using either rituximab alone (87 %) or combined with corticosteroid for the first 2 months showed disease response in 97 % of patients at month 6 [13]. Similar positive results from several smaller case series suggest that rituximab should be considered in cases refractory to corticosteroids and/or other disease-modifying antirheumatic drugs [14–16]. The authors suggest that B-cell depletion is an effective therapy in IgG4-associated disease, thereby supporting the use of rituximab. Rituximab depletes CD20-positive B cells that differentiate into plasma cells that produce IgG4 [14], and this can provide more specific, targeted therapy in patients with IgG4-associated disease.

Our first patient with necrotizing scleritis was refractory to oral corticosteroids and other immunomodulating agents such as methotrexate. Rituximab was not tried given that its possibility of therapeutic advantage was not known at the time of her treatment. The second patient underwent slow prednisone taper with a maintenance dose of methotrexate; however, the vision had minimal improvement despite the quiescent disease activity. Interestingly, only one of our patients (case 3) was treated with rituximab and had an excellent outcome. Even though rituximab was not an option due to insurance in case 2, this would have been our therapy of choice.

Even though IgG4-related inflammation affects many different organs, the histopathology is remarkably similar in most cases, and this is critical in making the diagnosis. It should be noted, however, that besides the typical fibro-inflammatory histologic picture generally seen in IgG4-related disease, associations between granulomatous inflammatory diseases and IgG4 are also being discovered, thereby broadening our understanding of the potential scope of IgG4-related inflammation. For example, cases of sarcoidosis, adult orbital xanthogranuloma, and granulomatosis with polyangiitis involving relatively high amounts of IgG4-positive plasma cells on histology and/or elevated IgG4 concentrations have all been recently reported [7, 17–20]. Interestingly, the necrotizing granulomatous inflammation on histology in our first case was evident only in the enucleated globe but not in the prior episcleral biopsy. The episcleral biopsy for the most part fit the typical histologic picture of IgG4-related disease, whereas the enucleated globe revealed the overlap between granulomatous sclero-uveitis and IgG4-related inflammation in this case. Accordingly, this case may be most properly diagnosed as granulomatosis with polyangiitis with an IgG4-related component. There are two other case reports of IgG4-related disease involving the episclera/sclera [3, 7]. In our case, the patient was much older than in the case reported by Ohno et al. [3], but both patients had a fibro-inflammatory mass lesion in the medial sclera. Their case underwent enucleation due to patient’s choice in the setting of diagnostic uncertainty, while ours progressed to a blind, painful eye despite aggressive immunosuppression. There was also a history of uveitis in their case, but unlike our case, no findings in the uvea were noted on histology of the enucleated globe. In a case reported by Paulus et al. [7], the patient was elderly, with a clinical presentation of sclero-uveitis and elevated p-ANCA, as in our case. The episcleral biopsy exhibited the typical histologic picture of IgG4-related disease, but unlike our case, the globe was not enucleated, so no histology of the uvea was available. A series of granulomatosis with polyangiitis cases reported by Chang et al. [20] found that cases involving the head and neck (sinonasal/orbital) were the ones most likely to exhibit an IgG4-related component on histology. Out of the six cases with elevated p-ANCA, four involved the sinonasal or orbital regions, and three of those four revealed an IgG4+/IgG+ plasma cell ratio of at least 30 %. It has also been discovered that many of the ANCA antibodies in patients with granulomatosis with polyangiitis are of the IgG4 subclass [21, 22].

Without the assistance of immunohistochemistry on histopathology, our second patient would have been diagnosed as having idiopathic hypertrophic pachymeningitis, and our third case would have been diagnosed as idiopathic orbital inflammatory pseudotumor. There are several reported cases of IgG4-related sclerosing pachymeningitis, and many of them were previously diagnosed as idiopathic hypertrophic pachymeningitis [23–25]. Lindstrom et al. [26] proposed a series of diagnostic criteria for IgG4-related meningeal disease: high serum IgG and/or IgG4 levels, infiltration of IgG4-positive plasma cells into the lesion, involvement of other organs possibly involved in IgG4-associated disease, exclusion of other diseases associated with high-serum IgG4 levels (e.g., atopic dermatitis, parasitic infections, pemphigus), exclusion of other diseases that could cause pachymeningitis, and good response to corticosteroids. Our case met the majority of diagnostic criteria except our patient had minimal response to steroids. Optic neuropathy in idiopathic hypertrophic pachymeningitis was shown to respond to therapy in six out of eight affected patients described by Kupersmith et al. [27]. Our patient may not have responded because of the duration and extent of the disease process prior to treatment.

IgG4-related disease is an idiopathic, multi-organ inflammatory state that can manifest as chronic, relapsing sclerosing inflammation in virtually any organ system. Ocular/orbital manifestations include idiopathic orbital inflammation, pachymeningitis, or sclero-uveitis. IgG4-associated disease is an important diagnosis to make given that it can guide more specific management. Making the diagnosis prevents further extensive diagnostic testing and multiple biopsies while allowing more specific and aggressive immunosuppression. Treatment may be different depending on the involved organ systems. Systemic corticosteroids are generally the first-line therapy currently, but rituximab can be combined or used as an alternative if patients have contraindications to corticosteroids; furthermore, a shift toward rituximab as first-line therapy may be seen in the future if more data on its success accumulate [28]. Physicians should be familiar with the diagnosis and be suspicious when patients present with chronic, sclerofibrosing inflammation with multi-systemic involvement. As the understanding of this entity increases, more specific diagnostic criteria and treatment will likely develop.

## Abbreviations

p-ANCA: Perinuclear accentuated anti-neutrophil cytoplasmic antibodies
BCVA: Best corrected visual acuity
MRI: Magnetic resonance imaging
OCT: Ocular coherence tomography
PCR: Polymerase chain reaction
